# Clinical performance of Abbott ID NOW™ COVID-19 2.0 rapid molecular point-of-care test compared to three real-time RT-PCR assays

**DOI:** 10.1128/spectrum.02033-24

**Published:** 2025-02-11

**Authors:** Maria D. Iglesias-Ussel, Aleah Bowie, Jack G. Anderson, Yin Li, Lawrence P. Park, Jose F. Cardona, Patrick Dennis, Valentine Ebuh, Steven A. Geller, Manish Jain, Mark M. McKenzie, Kian Merchant-Borna, Anand Patel, Amy Siegel, Guy S. Strauss, Joby J. Thoppil, Aaron S. Weinberg, Christopher W. Woods

**Affiliations:** 1 Division of Infectious Diseases, Department of Medicine, Duke University School of Medicine 3065 , Durham, North Carolina, USA; 2 Abbott Rapid Diagnostics, a Division of Abbott Laboratories, San Diego, California, USA; 3 Duke Global Health Institute, Duke University, Durham, North Carolina, USA; 4 Indago Research and Health Center Inc, Hialeah, Florida, USA; 5 DelRicht Research, New Orleans, Louisiana, USA; 6 Global Medical Research LLC, Dallas, Texas, USA; 7 Centennial Medical Group, Elkridge, Maryland, USA; 8 Great Lakes Clinical Trials, Chicago, Illinois, USA; 9 WR - ClinSearch, LLC, Chattanooga, Tennessee, USA; 10 Department of Emergency Medicine, University of Rochester School of Medicine and Dentistry 6927 , Rochester, New York, USA; 11 Conquest Research LLC, Winter Park, Florida, USA; 12 MediSync Clinical Research LLC, Austin, Texas, USA; 13 Multi-Specialty Research Associates Inc, Lake City, Florida, USA; 14 Department of Emergency Medicine, The University of Texas Southwestern Medical Center 12334 , Dallas, Texas, USA; 15 Carbon Health Technologies Inc.& Carbon Health Medical Group, Oakland, California, USA; National Microbiology Laboratory, Winnipeg, Manitoba, Canada

**Keywords:** COVID-19, SARS-CoV-2, isothermal, rRT-PCR, ID NOW, POC, rapid testing, nicking endonuclease amplification reaction (NEAR)

## Abstract

Timely diagnosis of SARS-CoV-2 is important for infection control and treatment. Real-time reverse transcriptase PCR (rRT-PCR) tests are the reference standard for diagnosis but often require a centralized laboratory, making them time-intensive and unsuitable for resource-limited settings. The Abbott ID NOW™ COVID-19 2.0 assay is a rapid point-of-care (POC), isothermal molecular test for qualitative detection of SARS-CoV-2. We prospectively evaluated its clinical performance against three reference rRT-PCR tests: Hologic Panther Fusion, Roche Cobas, and CDC 2019-nCoV RT-PCR Diagnostic Panel. Investigators enrolled 3,530 subjects, with 3,146 evaluable. In symptomatic subjects (*n* = 914), the test showed a positive percent agreement (PPA) of 91.7% (95% confidence interval [CI]: 87.8, 94.4) and a negative percent agreement (NPA) of 98.4% (95% CI: 97.1, 99.1). The PPA improved with lower cycle threshold (Ct) values: 94.7% (95% CI: 91.2, 97.2) for Ct ≤36, 97.6% (95% CI: 94.5, 99.2) for Ct ≤33, and 99.4% (95% CI: 96.8, 100.0) for Ct ≤30. Discordant results were observed among the three reference rRT-PCR tests across evaluable subjects with suspected COVID-19 infection. For 1,630 cases of symptomatic and asymptomatic subjects suspected of COVID-19, where all three rRT-PCR methods were evaluable, CDC test results differed the most, with 144 discordant results with Roche and 119 with Panther rRT-PCR tests. Roche and Panther test results differed in 67 cases. In summary, the Abbott ID NOW™ COVID-19 2.0 assay can serve as a valuable diagnostic tool in acute symptomatic subjects in point-of-care settings.

**IMPORTANCE:**

The Abbott ID NOW^TM^ COVID-19 2.0 assay is a suitable rapid test for diagnosing COVID-19 in acute symptomatic subjects and can be used in point-of-care settings and low-resource settings. With results reported in 12 minutes or less, Abbott ID NOW^TM^ COVID-19 2.0 facilitates timely diagnosis, enabling linkage to appropriate antiviral medication.

## INTRODUCTION

Severe acute respiratory syndrome coronavirus 2, commonly referred to as SARS-CoV-2, is an enveloped, positive-sense, single-stranded RNA virus (+ssRNA), causative of the coronavirus disease 2019 (COVID-19). SARS-CoV-2 belongs to the Coronaviridae family and the Sarbecovirus subgenus, which encompasses several other viruses capable of causing mild to severe infections in humans. SARS-CoV-2 primarily targets the lower respiratory tract and other tissues that express the ACE2 receptor. This virus exhibits a high level of transmissibility among humans (1) and has rapidly disseminated across the globe since 2019 through close contact or the expulsion of respiratory droplets (from coughing or sneezing) from infected individuals. On 11 March 2020, the Director-General of the World Health Organization (WHO) declared the COVID-19 outbreak a pandemic (2). Although no longer considered a global emergency (3), rapid diagnosis of COVID-19 is still crucial to mitigate transmission (4), facilitate early treatment, and reduce disease morbidity and mortality, especially among the vulnerable population, with higher-than-average risk of serious COVID-19 illness (5). Among the viral diagnostic tests, nucleic acid amplification tests (NAATs), detecting one or more viral ribonucleic acid (RNA) genes, are more sensitive than antigen/rapid/at-home/self-tests, Real-time reverse transcription polymerase chain reaction (rRT-PCR) tests provide high sensitivity and specificity. However, some rRT-PCR tests require sophisticated equipment and trained personnel, are expensive, and are most suitable for use in centralized diagnostic laboratories. Therefore, the turnaround time for obtaining results may be 1–2 days and can be further delayed due to increased demand. In contrast, antigen-based tests require minimal sample processing and provide results in minutes, making them a preferred approach for point-of-care (POC) and low-resource settings, but with a substantial reduction in test sensitivity (6). There remains a need for both rapid and accurate testing in near patient settings.

Abbott ID NOW^TM^ (7) is a POC isothermal NAAT using nicking endonuclease amplification reaction (NEAR) technology. The COVID-19 assay provides a qualitative result (positive, negative, or invalid) for SARS-CoV-2. The primers are designed to amplify a unique region of the SARS-CoV-2 RNA-dependent RNA polymerase (RdRp) gene segment. Results can be integrated automatically with medical records, which saves time and avoids errors. The Abbott ID NOW^TM^ COVID-19 version 2.0 had multiple updates from the previous version, such as chemistry changes that reduce invalid rates and the incorporation of a control with inactivated SARS-CoV-2 virus, providing results in 12 minutes or less with a limit of detection (LOD) of 500 copies/swab. We conducted a multicenter study to estimate the clinical positive percentage agreement (PPA) and negative percentage agreement (NPA) of the Abbott ID NOW™ COVID-19 2.0 assay against qualitative results from three rRT-PCR *in vitro* diagnostic tests, used as a reference to determine the Patient Infected Status (PIS): Hologic Panther Fusion SARS-CoV-2 Assay, Roche cobas SARS-CoV-2, and CDC 2019-Novel Coronavirus (2019-nCoV) real-time RT-PCR Diagnostic Panel. The first two tests have been introduced into the market under an emergency use authorization (EUA), and the CDC stopped the use of its 2019-nCoV Real-Time RT-PCR Diagnostic Panel after 31 December 2021.

The objective of this study was to estimate the clinical positive agreement and negative agreement of the Abbott ID NOW™ COVID-19 2.0 assay against three comparator methods in asymptomatic subjects and in subjects with symptoms of COVID-19 infection, using healthcare worker-collected nasal and nasopharyngeal swab specimens tested directly (i.e., without dilution in viral transport media).

Although numerous COVID-19 diagnostic tests have been developed, few of them are available in CLIA-waived and low-resource settings. In addition to clinical performance, turnaround time, ease of use, and cost, other important aspects are to be considered.

We provide results showing the Abbott ID NOW^TM^ COVID-19 2.0 assay is rapid, accurate in acute symptomatic subjects, and can be used in POC and low-resource settings.

The FDA granted Class II clearance and Clinical Laboratory Improvement Amendments of 1988 (CLIA) waiver for the ID NOW™ COVID-19 2.0 assay on 10 August 2023 for the qualitative detection of nucleic acids from SARS-CoV-2 in direct anterior nasal or nasopharyngeal swabs from individuals with signs and symptoms of respiratory tract infection. The test is authorized to be used in laboratories certified under CLIA and patient care settings operating under a CLIA Certificate of Waiver, Certificate of Compliance, or Certificate of Accreditation.

## MATERIALS AND METHODS

### Study design

Between December 2020 and January 2021, a total of 3,530 subjects were enrolled from 22 sites throughout the United States (Table S1). Institutional review board approval for the protocol “Clinical Evaluation of the Investigational ID NOW™ COVID-19 Assay” was obtained by each site. Enrollment included 1,920 subjects with suspected COVID-19 infection (with or without symptoms)—Cohort A—and 1,610 asymptomatic subjects with no reason to suspect COVID-19 exposure—Cohort B. Most subjects were enrolled in outpatient settings and urgent care sites, except for four subjects enrolled at a Nursing & Rehabilitation Center (Table S2, subject enrollment by setting).

### Abbott ID NOW^TM^ COVID-19 2.0

**Fig 1:**
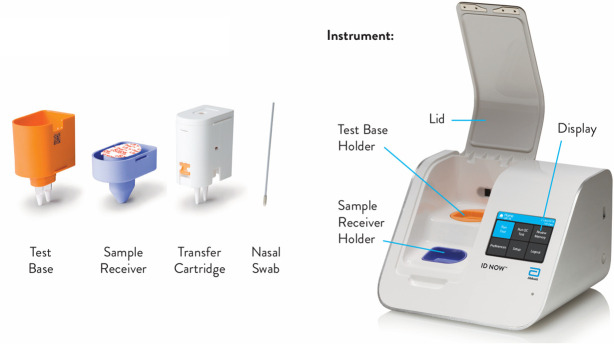
Abbott ID NOW™ platform. This platform comprises a Sample Receiver, containing elution/lysis buffer; a Test Base, comprising two sealed reaction tubes, each containing a lyophilized pellet; a Transfer Cartridge, for transfer of the eluted sample to the Test Base; and the ID NOW™ Instrument.

The Abbott ID NOW^TM^ platform comprises a Sample Receiver, containing elution/lysis buffer; a Test Base, comprising two sealed reaction tubes, each containing a lyophilized pellet; a Transfer Cartridge, for transfer of the eluted sample to the Test Base; and the ID NOW™ Instrument (Fig. 1). To perform the assay, the Sample Receiver and Test Base are inserted into the ID NOW™ Instrument. The sample is added to the Sample Receiver and transferred via the Transfer Cartridge to the Test Base, initiating target amplification. Heating, mixing, and detection are provided by the instrument.

The FDA granted Class II clearance and CLIA waiver for the ID NOW™ COVID-19 2.0 assay on 10 August 2023 for the qualitative detection of nucleic acids from SARS-CoV-2 in direct anterior nasal or nasopharyngeal swabs from individuals with signs and symptoms of respiratory tract infection. The test is authorized to be used in laboratories certified under Clinical Laboratory Improvement Amendments of 1988 and patient care settings operating under a CLIA Certificate of Waiver, Certificate of Compliance, or Certificate of Accreditation.

Moreover, the performance of the Abbott ID NOW™ COVID-19 2.0 assay is not anticipated to be impacted by the different circulating viral strains since this assay amplifies a segment of the RdRp gene, which is less susceptible to mutation than the spike gene (8).

### Sample collection and testing

Once written consent or assent was obtained, study staff collected either two anterior nasal swabs (NS) or two nasopharyngeal swabs (NPS) from subjects following standardized procedures (9). All subjects assigned an odd subject ID (i.e., 001, 003, 005, and 007) by study staff provided two NS, and all subjects assigned an even subject ID (i.e. 002, 004, 006, and 008) provided two NPS. Staff collected a single NPS from each nostril, whereas each NS was collected from both nostrils. To remove bias, the two nasal swabs were collected concurrently and then switched to the second nostril. One of the two collected NS or NPS was tested as soon as possible after collection (or within an hour of being stored at room temperature) and without elution in Universal Transport Media (UTM) using the ID NOW™ COVID-19 2.0 assay. To ensure the study design incorporated the ability to test the ease-of-use of the assay as well as the system, sites that had no previous training on the ID NOW™ platform were specifically identified. The other swab was immediately (within 10 minutes) eluted in UTM, and the swab head was left in the media tube for comparator testing. UTM samples were stored at 2°C–8°C and sent in cold packs daily to the central laboratory for reference testing.

The workflow in the central lab processed the eluted UTM swab samples on three comparator laboratory rRT-PCR *in vitro* diagnostic tests: Hologic Panther Fusion SARS-CoV-2 Assay, Roche cobas SARS-CoV-2, and CDC 2019-Novel Coronavirus (2019-nCoV) real-time RT-PCR Diagnostic Panel, according to the manufacturer’s instructions for use, before adjudicating infection status. Specimens received ≥72 hours after collection were considered unevaluable, unless they were shipped frozen. Each day, prior to subject specimen testing, site study staff performed external control testing on each instrument using positive and negative control swabs.

### Case definition Patient Infected Status

PIS is a composite reference classification based on the qualitative results of the eluted UTM samples on the three real-time RT-PCR *in vitro* diagnostic tests. In the cases where the qualitative result from the first two comparator tests differed, or one of the first two comparator tests did not have a valid result, the third test (CDC 2019-nCoV) determined the PIS (Table 3). CDC results of “Inconclusive” are handled as a special case in Table 3. In cases with discordant qualitative results between the first two comparator tests (one positive and one negative), an inconclusive CDC 2019-nCoV result (indicating that one of two targets is detected) determines PIS status as positive (PIS+).

### Data analysis

Positive percent agreement and negative percent agreement were calculated based on two-by-two contingency tables, along with corresponding 95% confidence intervals, calculated using the exact method for a binomial proportion. Statistical analysis was performed in SAS and MATLAB. Results were stratified by presence and duration of symptoms, by sample type (NS and NPS), and by cohort. Positive agreement between the ID NOW™ COVID-19 test and PIS was also stratified by rRT-PCR Ct values (Ct <30, Ct <33, and Ct <36).

The ID NOW™ COVID-19 test was also compared to each of the three individual tests used for reference (Panther, Roche, and CDC). In addition, the three tests used for reference were compared among themselves in pairs.

## RESULTS

**Fig 2:**
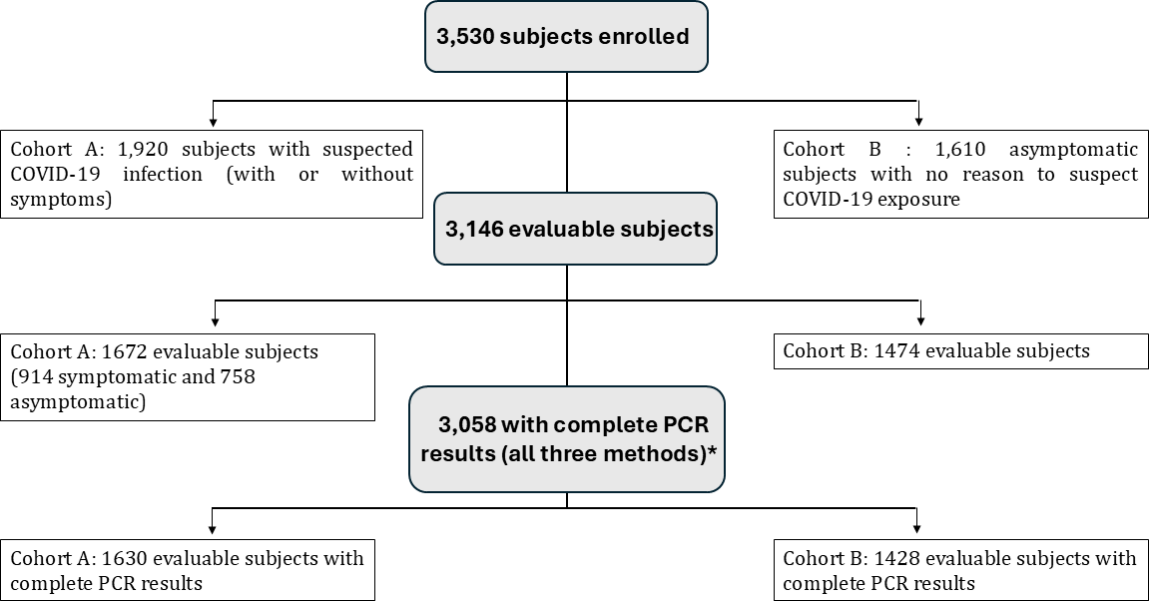
Subject disposition by Cohort. *A total of 88 subjects with incomplete PCR results on one of three methods (60 subjects without Panther, 27 subjects without Roche, and 1 subject without CDC (but PIS was still fully determined).

Of the 3,530 subjects enrolled over the study period (December 2020 through January 2021), 3,146 (89%) were determined evaluable (Table S3): 1,672 with suspected COVID-19 infection (with or without symptoms) (Cohort A), and 1,474 asymptomatic subjects with no reason to suspect COVID-19 exposure (Cohort B) (Fig. 2). A total of 384 subjects were not evaluable for a variety of reasons, such as protocol deviations, withdrawal, and invalid results, as summarized in Table S3, and were thus excluded from analyses. Of the 3,146 evaluable subjects, 3,058 had complete rRT-PCR results (all three methods) and 88 had incomplete rRT-PCR results on one of the three methods (60 subjects without Panther, 27 subjects without Roche, and 1 subject without CDC). There were 105 evaluable fresh specimens received between 48 hours and 72 hours after collection. Although they were outside the recommended time for the Roche method, they were within the recommended time for the other two PCR methods, and only nine of these depended on the Roche result for the determination of PIS (Table S4).

Demographics for the evaluable subjects are presented in Table 1 by cohort. The median age of patients was 37 years, with slightly more women than men. The majority of subjects (75.7%) were white, followed by black (16.2%) subjects. Symptoms at presentation among symptomatic subjects are summarized in Table 2.

**TABLE 1:** Subject demographics by cohort^*a*^

	All evaluable (*N* = 3,146)	Cohort A (*N* = 1,672)	Cohort SYMP (*N* = 914)	Cohort B (*N* = 1,474)
Age				
N	3,146	1,672	914	1,474
Mean (SD)	39 (18.0)	40 (18.1)	42 (17.3)	38 (17.8)
Median	37	39	40	35
Min–Max	0.7, 90	0.7, 90	1.0, 87	0.9, 86
Gender				
F	1,846 (58.7%)	963 (57.6%)	560 (61.3%)	883 (59.9%)
M	1,300 (41.3%)	709 (42.4%)	354 (38.7%)	591 (40.1%)
Race				
Asian	58 (1.8%)	22 (1.3%)	17 (1.9%)	36 (2.4%)
Black	509 (16.2%)	210 (12.6%)	98 (10.7%)	299 (20.3%)
Pacific Islander	8 (0.3%)	5 (0.3%)	3 (0.3%)	3 (0.2%)
Mixed Race	28 (0.9%)	12 (0.7%)	6 (0.7%)	16 (1.1%)
Native American	10 (0.3%)	1 (0.1%)	1 (0.1%)	9 (0.6%)
No answer	150 (4.8%)	69 (4.1%)	33 (3.6%)	81 (5.5%)
White	2,383 (75.7%)	1,353 (80.9%)	756 (82.7%)	1,030 (69.9%)
Ethnicity				
Hispanic	794 (25.2%)	444 (26.6%)	281 (30.7%)	350 (23.7%)
No answer	126 (4.0%)	73 (4.4%)	29 (3.2%)	53 (3.6%)
Not Hispanic	2,226 (70.8%)	1,155 (69.1%)	604 (66.1%)	1,071 (72.7%)

^*a*^ Cohort A: subjects with suspected COVID-19 infection (with or without symptoms); Cohort B: asymptomatic subjects with no reason to suspect COVID-19 exposure.

**TABLE 2:** Medical history and symptoms at presentation^*a*^

	Symptoms at presentation among evaluable subjects, N (%) All (*N* = 3,146)	Cohort SYMP (*N* = 914)
Nursing home resident		
N	3,141 (99.8%)	911 (99.7%)
Y	5 (0.2%)	3 (0.3%)
Previously received a positive COVID-19 result		
N	2,803 (89.1%)	781 (85.4%)
Y	343 (10.9%)	133 (14.6%)
Hospital inpatient		
ADMIT	7 (0.2%)	2 (0.2%)
NOT	3,139 (99.8%)	912 (99.8%)
Symptom days		
N	913	913
Mean (SD)	4.1 (4.9)	4.1 (4.9)
Median	3.0	3.0
Min–Max	0.0, 90	0.0, 90
Diarrhea		
No	2,978 (94.7%)	746 (81.6%)
Yes	168 (5.3%)	168 (18.4%)
Cough		
No	2,633 (83.7%)	401 (43.9%)
Yes	513 (16.3%)	513 (56.1%)
Tiredness		
No	2,787 (88.6%)	555 (60.7%)
Yes	359 (11.4%)	359 (39.3%)
Fever		
No	2,979 (94.7%)	747 (81.7%)
Yes	167 (5.3%)	167 (18.3%)
Headache		
No	2,636 (83.8%)	404 (44.2%)
Yes	510 (16.2%)	510 (55.8%)
Loss of taste		
No	2,988 (95.0%)	756 (82.7%)
Yes	158 (5.0%)	158 (17.3%)
Muscle ache		
No	2,739 (87.1%)	507 (55.5%)
Yes	407 (12.9%)	407 (44.5%)
Runny nose		
No	2,661 (84.6%)	429 (46.9%)

^*a*^ One subject had onset of an unknown symptom and was excluded from the analysis.

### Abbott ID NOW™ COVID-19 clinical performance

#### Symptomatic subjects

The clinical performance of the ID NOW™ COVID-19 2.0 assay was evaluated by estimating the clinical PPA and NPA of the ID NOW™ COVID-19 2.0 assay against the PIS, determined by testing the NS or NPS eluted in UTM on comparator tests (Table 3). For symptomatic subjects, 277 were PIS positive (PIS+), and 254 were positive by the ID NOW™ COVID-19 2.0 assay. Therefore, the ID NOW™ COVID-19 2.0 assay demonstrated an overall PPA of 91.7% (95% CI: 87.8, 94.7) and an overall NPA of 98.4% (95% CI: 97.1, 99.2) for symptomatic individuals. Performance was similar regardless of the sample type, with a PPA of 93.2% (95% CI: 87.8, 96.7) for NPS and 90.0% (95% CI: 83.5, 94.6) for NA and an NPA of 98.4% (95% CI: 96.2, 99.) and 98.5% (95% CI: 96.5, 99.5) for NPS and NS, respectively (Table 4).

**TABLE 3:** Patient Infection Status designation^*a*^

Comparator 1 (Panther)	Comparator 2 (Roche)	Comparator 3 (CDC)	PIS
Positive	Positive	NA	Positive
Positive	Presumptive positive	NA	Positive
Positive	Negative	Positive	Positive
Positive	Negative	Negative	Negative
Positive	Negative	Inconclusive	Positive
Positive	Negative	Invalid	Inconclusive
Positive	Invalid	Positive	Positive
Positive	Invalid	Negative	Inconclusive
Positive	Invalid	Inconclusive	Inconclusive
Positive	Invalid	Invalid	Invalid
Negative	Negative	NA	Negative
Negative	Positive	Positive	Positive
Negative	Positive	Negative	Negative
Negative	Positive	Inconclusive	Positive
Negative	Positive	Invalid	Inconclusive
Negative	Presumptive positive	Positive	Positive
Negative	Presumptive positive	Negative	Negative
Negative	Presumptive positive	Inconclusive	Inconclusive
Negative	Presumptive positive	Invalid	Inconclusive
Negative	Invalid	Positive	Inconclusive
Negative	Invalid	Negative	Negative
Negative	Invalid	Inconclusive	Inconclusive
Negative	Invalid	Invalid	Invalid
Invalid	Positive	Positive	Positive
Invalid	Positive	Negative	Inconclusive
Invalid	Positive	Inconclusive	Inconclusive
Invalid	Positive	Invalid	Inconclusive
Invalid	Presumptive positive	Positive	Positive
Invalid	Presumptive Positive	Negative	Inconclusive
Invalid	Presumptive Positive	Inconclusive	Inconclusive
Invalid	Presumptive Positive	Invalid	Invalid
Invalid	Negative	Positive	Inconclusive
Invalid	Negative	Negative	Negative
Invalid	Negative	Inconclusive	Inconclusive
Invalid	Negative	Invalid	Invalid
Invalid	Invalid	NA	Invalid

^*a*^ NA represents the cases when Comparator 3 is not required to determine PIS because the qualitative result from the first two comparator tests did not differ, e.g., Panther Positive with Roche Positive (or Presumptive Positive) or Panther Negative with Roche Negative.

**TABLE 4:** Agreement between the ID NOW™ COVID-19 2.0 test and PIS in symptomatic subjects^*a*^

	Symptomatic (NPS)			Symptomatic (NS)			Symptomatic (NPS and NS)		
**ID-NOW™**	**PIS +**	**PIS –**	**Total**	**PIS +**	**PIS –**	**Total**	**PIS +**	**PIS –**	**Total**
Positive	117	5	122	137	5	142	254	10	264
Negative	13	325	338	10	302	312	23	627	650
Total	130	330	460	147	307	454	277	637	914
PPA (95% CI) (%)	90.0 (83.5, 94.6)			93.2 (87.8, 96.7)			91.7 (87.8, 94.4)		
NPA (95% CI) (%)	98.5 (96.5, 99.5)			98.4 (96.2, 99.5)			98.4 (97.1, 99.1)		

^*a*^ NPS: nasopharyngeal swab; NS: nasal swab.

When looking at the prevalence of confirmed COVID-19 cases with symptomatic subjects (Table 5), there were 277 PIS +subjects (prevalence 30.3%), of which one PIS +subject (prevalence 33.3%) was≤5 years of age, 37 PIS +subjects (prevalence 33.3%) were 6–21 years old, 191 PIS +subjects had were 22–59 years old (prevalence 29.7%), and 48 PIS +subjects were found in the ≥60 years category (prevalence 30.8%).

**TABLE 5:** Prevalence of confirmed COVID-19 cases, based on the proportion of PIS +subjects, for the different age categories within symptomatic subjects

Age	Total	PIS+	Prevalence
≤5	3	1	33.3%
6–21	111	37	33.3%
22–59	644	191	29.7%
≥ 60	156	48	30.8%
All	914	277	30.3%

#### Asymptomatic subjects

The PPA was 82.9% (95% CI: 67.9, 92.8) in 41 PIS positive (PIS+) asymptomatic subjects with no reason to suspect COVID-19 exposure and screened for several reasons (Cohort B) and 69.0% (95% CI: 55.5, 80.5) in 58 PIS +asymptomatic subjects with suspected COVID-19 infection by their healthcare provider (from Cohort A). However, the test showed high specificity in these subjects since the NPA was 98.4% (95% CI: 97.2, 99.2) in 700 PIS negative (PIS–) asymptomatic subjects with suspected COVID-19 infection (from Cohort A) and 98.7% (95% CI: 97.9, 99.2) in 1,433 PIS– asymptomatic subjects without suspected exposure (Cohort B).

### Performance relative to assigned Ct value

**Fig 3:**
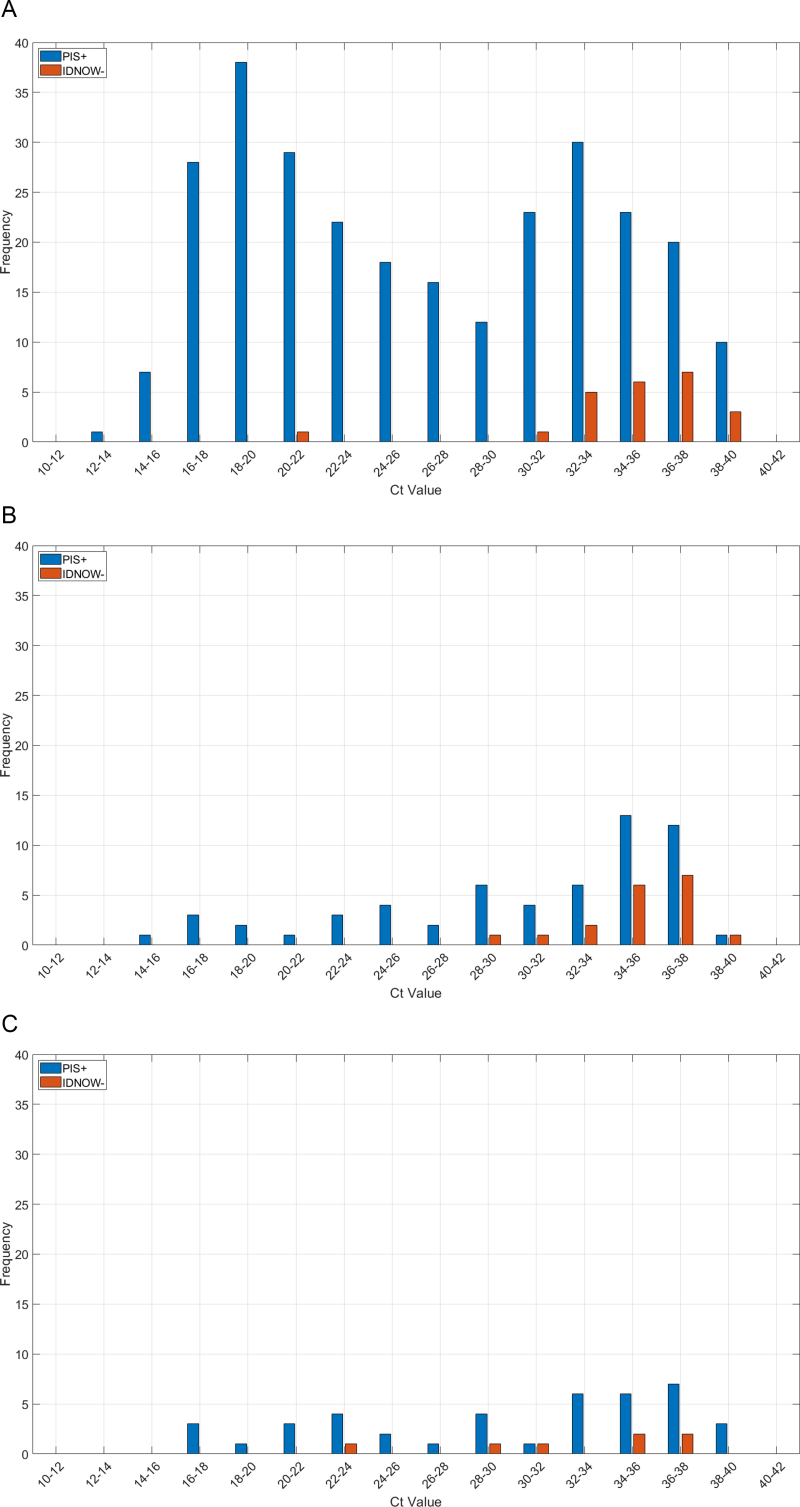
Distribution of Ct values in the PIS +subjects together with discordant results (ID NOW negative) for: (**A**) symptomatic subjects (*N* = 277), (**B**) asymptomatic subjects with suspected COVID-19 infection (*N* = 58), and (**C**) asymptomatic subjects with no reason to suspect COVID-19 exposure (*N* = 41).

We then looked at the distribution of Ct values among ID NOW™ COVID-19 2.0 negative but PIS +discordant cases in symptomatic subjects (Fig. 3A), as well as asymptomatic subjects with suspected COVID-19 infection (Fig. 3B) and asymptomatic subjects with no reason to suspect exposure to COVID-19 (Fig. 3C). A single Ct value was assigned to each PIS +sample. The Ct value was assigned by priority, following the PIS definition in Table 3. Therefore, first priority was given to the Panther Ct value, and Roche Ct value was assigned at second priority (when the Panther result was negative). For Roche, further priority was given to the Ct value for target 1 and then the Ct value for target 2.

The frequency of discordant results between ID NOW™ COVID-19 2.0 and comparator tests increased mostly for Ct values ≥ 30 (Fig. 3A through C).

Furthermore, we stratified the sensitivity of the ID NOW™ COVID-19 2.0 test according to Ct values for PIS +subjects. The overall PPA for symptomatic subjects for both NS and NPS combined increased from 91.7% (95% CI: 87.8, 94.7) for Ct values of up to 40 (all PIS +subjects) to 94.7% (95% CI: 91.2, 97.2) for Ct values up to 36, to 97.6% (95% CI: 94.5, 99.2) for

Ct values up to 33, and to 99.4% (95% CI: 96.8, 100.0) for Ct values up to 30 (Table 6).

**TABLE 6:** Agreement between the ID NOW™ COVID-19 2.0 test and PIS at various PCR cycle thresholds within symptomatic subjects

Cohort A, symptomatic subjects				
	PIS +All Ct	Ct <30	Ct <33	Ct <36
ID NOW Positive	254	170	204	234
ID NOW Negative	23	1	5	13
Total	277	171	209	247
PPA (95% CI)	91.7 (87.8, 94.7)	99.4 (96.8, 100.0)	97.6 (94.5, 99.2)	94.7 (91.2, 97.2)

In asymptomatic subjects with suspected COVID-19 infection, the PPA increased from 69.0% (CI: 55.5, 80.5) to 77.8% (CI: 62.9, 88.8) for Ct values up to 36, to 92.9% (CI: 76.5, 99.1) for Ct values up to 33, and to 95.5% (CI: 77.2, 99.9) for Ct values up to 30 (Table S5).

### Discordant results among tests

Pairwise method comparisons were used to determine differences in the results of all four tests across all evaluable subjects in Cohort A (*N* = 1,672) (Table 7). Roche presumptive positive results were treated as positive, and CDC inconclusive results were treated as positive for this analysis. Out of the 1,672 evaluable subjects in Cohort A, 1,630 had results with all PCR tests.

**TABLE 7:** Discordant results by test^*a*^

Cohort A, all evaluable subjects with complete PCR							
Method Comparison	X + Y +	X + Y–	X–Y+	X–Y–	N	PPA	NPA
ID NOW (X) vs PIS (Y)	288	21	40	1,281	1,630	87.8%	98.4%
ID NOW (X) vs PANTHER (Y)	286	23	40	1,281	1,630	87.7%	98.2%
ID NOW (X) vs ROCHE (Y)	285	24	66	1,255	1,630	81.2%	98.1%
ID NOW (X) vs CDC (Y)	287	22	114	1,207	1,630	71.6%	98.2%
PANTHER (X) vs ROCHE (Y)	310	16	41	1,263	1,630	88.3%	98.7%
ROCHE (X) vs PANTHER (Y)	310	41	16	1,263	1,630	95.1%	96.9%
PANTHER (X) vs CDC (Y)	304	22	97	1,207	1,630	75.8%	98.2%
CDC (X) vs PANTHER (Y)	304	97	22	1,207	1,630	93.3%	92.6%
ROCHE (X) vs CDC (Y)	304	47	97	1,182	1,630	75.8%	96.2%
CDC (X) vs ROCHE (Y)	304	97	47	1,182	1,630	86.6%	92.4%

^*a*^ There were 1,672 evaluable subjects in Cohort A, but 42 subjects were excluded due to one of the three comparator methods being unevaluable, leaving 1,630 subjects for this analysis.

When compared to the composite reference classification of the three rRT-PCR tests used for PIS, the ID NOW™ COVID-19 2.0 assay gave discordant results in 61 out of 1,630 cases since 42 subjects were excluded due to one of the three comparator methods being unevaluable. The biggest difference was observed between the ID NOW™ COVID-19 2.0 and the CDC test, with 136 discordant results. ID NOW™ COVID-19 2.0 and Roche were discordant in 90 cases and ID NOW™ COVID-19 2.0 and Panther in 63.

Differences were also observed among the three real time RT-PCR tests used for reference. CDC test results differed the most with the other two rRT-PCR tests, with 144 discordant results with Roche and 119 with Panther’s test. Roche and Panther test results differed in 67 cases.

## DISCUSSION

In this multicenter registrational study, we evaluated the clinical performance characteristics of the Abbott ID NOW™ COVID-19 2.0 assay against the qualitative results of three rRT-PCR SARS-CoV-2 assays (Panther Fusion, Roche Cobas, and the now retired CDC 2019-nCoV), used as a composite reference to determine infected status.

The Abbott ID NOW™ COVID-19 2.0 rapid isothermal nucleic acid amplification assay had a high agreement with rRT-PCR SARS-CoV-2 comparator tests, particularly regarding specificity (NPA) in symptomatic subjects, regardless of whether anterior NS or NPS were used.

Clinical sensitivity of NAATs is affected by the assay’s limit of detection (LOD); therefore, sensitivity is usually higher during the acute period of infection, when the viral load is the highest in the respiratory tract (10). SARS-CoV-2 RNA shedding in the upper respiratory tract can last for prolonged periods and be detected by RT-PCR for a mean of 17 days after symptom onset (11). However, a positive test result late in the course of the infection does not necessarily indicate a person is infectious or that symptoms are driven by viral replication. Indeed, rRT-PCR does not differentiate between viable and nonviable viruses, but live virus can rarely be isolated beyond 9 days of symptoms (12, 13).

Lower PPAs were observed in asymptomatic subjects, although the clinical sensitivity of NAAT tests is usually lower in asymptomatic people than in symptomatic people (14). PPA was slightly higher (82.9%) in asymptomatic subjects with no reason to suspect exposure to COVID-19 than in asymptomatic subjects with suspected COVID-19 infection (69.0%), although the sample size of PIS +subjects in both asymptomatic populations was small (41 and 58, respectively). Although the test had a good NPA in these subjects, it is important to note that this test is not authorized for use in the asymptomatic population.

Discordant results, or negative Abbott ID NOW™ COVID-19 2.0 test results with a PIS +reference, were observed for assigned Ct values above 30 on comparator rRT-PCR tests. As mentioned previously, a single Ct value was assigned to each PIS +sample following the priority rules of PIS (Panther Ct value, Roche target 1 Ct, and Roche target 2 Ct). The Ct value, or cycle threshold, corresponds to the PCR cycle number at which the fluorescence generated within the reaction crosses the fluorescence background. Therefore, the Ct is inversely proportional to the original relative amount of target nucleic acids in the sample, in this case as a proxy for viral load (15). Thus, it is expected for the sensitivity required to detect SARS-CoV-2 to decrease as the viral load decreases. Ct thresholds of up to 33 cycles rendered improved PPAs in both symptomatic subjects and asymptomatic subjects. It has been reported that virus propagation decreases as Ct values increase (16) and subjects with Ct values above 33 may no longer be infectious. However, Ct values across different rRT-PCR platforms are not standardized, and Ct values are currently not being used to guide clinical management of infected subjects.

When ID NOW™ COVID-19 2.0 results in evaluable subjects, with suspected COVID-19 infection by their healthcare provider, were compared to the those of the three rRT-PCR tests used for reference, the most discordant results were observed with the CDC test, followed by Roche’s, and then the Panther test. Discordant results were also observed among the three rRT-PCR tests used for reference. The greatest difference was observed between Roche and CDC tests, then between Panther and CDC, and then between Roche and Panther. These discrepancies highlight there is no single gold-standard molecular test, so clinical judgement should always be considered part of the diagnosis.

Numerous COVID-19 diagnostic tests have been developed and granted marketing authorization by the FDA, although few are available in CLIA waived settings. In addition to clinical performance, there are other important aspects to consider, such as turnaround time, ease of use, and cost. The Abbott ID NOW^TM^ COVID-19 2.0 assay is rapid, accurate in acute symptomatic subjects, and can be used in POC and low-resource settings.

## SUPPLEMENTAL MATERIAL

Supplemental tables spectrum.02033-24-s0001.docx Tables S1 to S5.

ASM does not own the copyrights to Supplemental Material that may be linked to, or accessed through, an article. The authors have granted ASM a non-exclusive, world-wide license to publish the Supplemental Material files. Please contact the corresponding author directly for reuse.

## References

[1] Elrashdy F, Redwan EM, Uversky VN. 2020. Why COVID-19 transmission is more efficient and aggressive than viral transmission in previous coronavirus epidemics? Biomolecules 10:1312. doi:10.3390/biom10091312 32933047 PMC7565143

[2] WHO director-general’s opening remarks at the media briefing on COVID-19. 2020. https://www.who.int/director-general/speeches/detail/who-director-general-s-opening-remarks-at-the-media-briefing-on-covid-19---11-march-2020.

[3] Roknuzzaman A, Sarker R, Shahriar M, Mosharrafa RA, Islam MR. 2024. The WHO has declared COVID-19 is no longer a pandemic-level threat: a perspective evaluating potential public health impacts. Clin Pathol 17:2632010X241228053. doi:10.1177/2632010X241228053 PMC10804921 38264675

[4] Peeling RW, Heymann DL, Teo YY, Garcia PJ. 2022. Diagnostics for COVID-19: moving from pandemic response to control. Lancet 399:757–768. doi:10.1016/S0140-6736(21)02346-1 34942102 PMC8687671

[5] Who is at high risk for severe coronavirus disease? 2024. Johns Hopkins Medicine. Available from: https://www.hopkinsmedicine.org/health/conditions-and-diseases/coronavirus/coronavirus-and-covid19-who-is-at-higher-risk

[6] Dinnes J, Sharma P, Berhane S, van Wyk SS, Nyaaba N, Domen J, Taylor M, Cunningham J, Davenport C, Dittrich S, Emperador D, Hooft L, Leeflang MM, McInnes MD, Spijker R, Verbakel JY, Takwoingi Y, Taylor-Phillips S, Van den Bruel A, Deeks JJ, Cochrane COVID-19 Diagnostic Test Accuracy Group. 2022. Rapid, point-of-care antigen tests for diagnosis of SARS-CoV-2 infection. Cochrane Database Syst Rev 7:CD013705. doi:10.1002/14651858.CD013705.pub3 35866452 PMC9305720

[7] Abbott point of care. 2024. Available from: https://www.globalpointofcare.abbott/us/en/product-details/id-now.html. Retrieved 5 Dec 2024.

[8] Abbott. 2023. Predicted impact of variants on Abbott’s SARS-CoV-2/COVID-19 diagnostic tests-technical brief

[9] Interim guidelines for collecting and handling of clinical specimens for COVID-19 testing | COVID-19 | CDC. 2024. Available from: https://www.cdc.gov/covid/hcp/clinical-care/clinical-specimen-guidelines.html. Retrieved 5 Dec 2024.

[10] Cevik M, Kuppalli K, Kindrachuk J, Peiris M. 2020. Virology, transmission, and pathogenesis of SARS-CoV-2. BMJ 371:m3862. doi:10.1136/bmj.m3862 33097561

[11] Cevik M, Tate M, Lloyd O, Maraolo AE, Schafers J, Ho A. 2021. SARS-CoV-2, SARS-CoV, and MERS-CoV viral load dynamics, duration of viral shedding, and infectiousness: a systematic review and meta-analysis. Lancet Microbe 2:e13–e22. doi:10.1016/S2666-5247(20)30172-5 33521734 PMC7837230

[12] Owusu D, Pomeroy MA, Lewis NM, Wadhwa A, Yousaf AR, Whitaker B, Dietrich E, Hall AJ, Chu V, Thornburg N, et al.. 2021. Persistent SARS-CoV-2 RNA shedding without evidence of infectiousness: a cohort study of individuals with COVID-19. J Infect Dis 224:1362–1371. doi:10.1093/infdis/jiab107 33649773 PMC7989388

[13] Arons MM, Hatfield KM, Reddy SC, Kimball A, James A, Jacobs JR, Taylor J, Spicer K, Bardossy AC, Oakley LP, et al.. 2020. Presymptomatic SARS-CoV-2 infections and transmission in a skilled nursing facility. N Engl J Med 382:2081–2090. doi:10.1056/NEJMoa2008457 32329971 PMC7200056

[14] Nucleic acid amplification testing (e.g. RT-PCR). 2023. Available from: https://www.idsociety.org/covid-19-real-time-learning-network/diagnostics/RT-pcr-testing

[15] Real-time PCR: understanding Ct - US.

[16] Singanayagam A, Patel M, Charlett A, Lopez Bernal J, Saliba V, Ellis J, Ladhani S, Zambon M, Gopal R. 2020. Duration of infectiousness and correlation with RT-PCR cycle threshold values in cases of COVID-19, England, January to May 2020. Euro Surveill 25:1. doi:10.2807/1560-7917.ES.2020.25.32.2001483 PMC7427302 32794447

