# Supplementary material for: Clinical performance of Abbott ID NOW™ COVID-19 2.0 rapid molecular point-of-care test compared to three real-time RT-PCR assays
Source: Microbiol Spectr. 2025 Feb 11;13(3):e02033-24. doi: 10.1128/spectrum.02033-24 (PMC11877999; doi:10.1128/spectrum.02033-24)
Supplement: Supplemental tables — Tables S1 to S5. [file spectrum.02033-24-s0001.docx]

**SUPPLEMENTAL MATERIAL**

**Supplemental Tables**

**Table S1** List of Principal Investigators and study sites

| **Principal Investigator** | **Site** |
| --- | --- |
| Jeff Harris, MD | Urgent Care Clinical Trials, LLC (UCCT) - Easley, SC  5208 Calhoun Memorial Hwy, Suite C Easley, SC 29640 |
| Christopher Woods, MD | Duke University 101 Science Dr., CIEMAS Building, Room 2149 Durham, NC 27710 |
| Natasha Ballard, MD | Urgent Care Clinical Trials (UCCT) - Chattanooga, TN  1521 Gunbarrel Road, Suite 103  Chattanooga, TN 37421 |
| Steven Geller, MD | Centennial Medical Group / First Call Urgent Care  8186 Lark Brown Road, Suite 201  Elkridge, MD 21075 |
| Patrick Dennis, MD | DelRicht Research 826 Harrison Ave  New Orleans, LA 70124 |
| Jose Cardona, MD | Indago Research and Health Center 3700 West 12 Ave., Suite 300  Hialeah, FL 33012 |
| Luis A. Martinez, MD | Universal Axon Clinical Research 3650 NW 82nd Avenue, Suite 503  Doral, FL 33166 |
| Manish Jain, MD | Great Lakes Clinical Trials  5149 North Ashland Ave. Chicago, IL 60640 |

| Aaron S. Weinberg, MD | Carbon Health Tech 411 Grande Avenue  Oakland, CA 94610 |
| --- | --- |
| Joby J. Thoppil, MD | UT Southwestern Medical Center 5323 Harry Hines Boulevard, Suite E4.300 Dallas, TX 75390 |
| Kian Merchant-Borna, MPH, MBA | University of Rochester Medical Center 601 Elmwood Ave, Box 655C, Rochester, NY 14642 |
| Clint Guillory, MD | Clinical Trials of America 3201 Armand St  Monroe, LA 71201 |
| Valentine Ebuh, MD | Global Medical Research, LLC 2701 South Hampton Road, Suite 250 Dallas, TX 75224 |
| Vicki James, MD | Essential Medical Care  5100 Old Bill Cook Road College Park, GA 30449 |
| Amy Siegel, MD | MediSync Clinical Research, LLC 111 West William Cannon Drive, Suite 312 Austin, TX 78745 |
| Adi Fatakia, MD | Tandem Clinical Research 1111 Medical Center Boulevard, Suite N308 Marrero, LA 70072 |
| Jared Probst, MD | Cognitive Clinical Trials - Olympus Family Medicine  4624 Holladay Boulevard  Holladay, UT 84117 |
| John Hemmersmeier, MD | Cognitive Clinical Trials - South Ogden Family Clinic  5740 Crestwood Dr  Ogden, UT 84405 |
| Guy S. Strauss, DO | Multi-Specialty Research Associates, Inc. |

|  | 4601 West US Highway 90,  Lake City, FL 32055 |
| --- | --- |
| Anand Patel, MD | Conquest Research, LLC 2233 Lee Road, Suite 101 Winter Park, FL 32789 |
| Mark M. McKenzie, MD | WR – ClinSearch, LLC  6035 Shallowford Road, Suite 109,  Chattanooga, TN 37421 |
| Prasad Padala, MD | Atria Clinical Research 11321, I-30, Suite 308 Little Rock, AR 72209 |

**Table S2** Subject enrollment by setting

| **Type of Setting** | **Cohort A** | **Cohort B** | **Total** |
| --- | --- | --- | --- |
| Nursing | 1 | 3 | 4 |
| Outpatient | 1627 | 1476 | 3103 |
| Urgent Care | 292 | 131 | 423 |
| **Total** | **1920** | **1610** | **3530** |

Cohort A: subjects with suspected COVID-19 infection (with or without symptoms); Cohort B: asymptomatic subjects with no reason to suspect COVID-19 exposure.

**Table S3** Reasons for unevaluable

|  | **All (N=3530)** | **Cohort A (N=1920)** | **Cohort B (N=1610)** |
| --- | --- | --- | --- |
| Not met inclusion criteria | 1 (0.0%) | 0 (0.0%) | 1 (0.1%) |
| Withdrawal | 7 (0.2%) | 0 (0.0%) | 7 (0.4%) |
| Invalid ID NOW^TM^ 2.0 result | 19 (0.5%) | 5 (0 3%) | 14 (0.9%) |
| Test time outside 80 min window | 3 (0.1%) | 2 (0.1%) | 1 (0.1%) |
| Sample not received at Lab | 10 (0.3%) | 6 (0.3%) | 4 (0.2%) |
| Sample mislabeled by Site | 3 (0 1%) | 2 (0.1%) | 1 (0.1%) |
| Sample leaked, QNS for reference test | 94 (2.7%) | 58 (3.0%) | 36 (2.2%) |
| Out of stability, no reference test | 176 (5.0%) | 116 (6.0%) | 60 (3.7%) |
| Sample shipment delayed > 72 hours | 57 (1.6%) | 52 (2.7%) | 5 (0 3%) |
| Sample not refrigerated prior to shipment | 3 (0.1%) | 0 (0.0%) | 3 (0.2%) |
| Inconclusive | 1 (0.0%) | 1 (0.1%) | 0 (0.0%) |
| Subject did not sign consent form | 7 (0.2%) | 3 (0.2%) | 4 (0.2%) |
| Due to technical issues, site only has a partial ICF | 1 (0.0%) | 1 (0.1%) | 0 (0.0%) |
| Parent-child aliquot labeling error | 2 (0 1%) | 2 (0.1%) | 0 (0.0%) |

Cohort A: subjects with suspected COVID-19 infection (with or without symptoms); Cohort B: asymptomatic subjects with no reason to suspect COVID-19 exposure.

Note: Four withdrawal subjects without symptom status were assigned to Cohort B.

**Table S4** Test results for specimens from evaluable subjects received outside the recommended time for the Roche method and relying on the Roche test result for PIS calculation

| **Subject ID** | **Cohort** | **STATUS** | **IDNOW** | **PANTHER** | **ROCHE** | **CDC** | **PIS** |
| --- | --- | --- | --- | --- | --- | --- | --- |
| 143A-065 | A | SYMPTOMATIC | NEGATIVE | NEGATIVE | NEGATIVE | INCONCLUSIVE | NEGATIVE |
| 143A-079 | B | ASYMPTOMATIC | NEGATIVE | NEGATIVE | NEGATIVE | POSITIVE | NEGATIVE |
| 143A-123 | A | ASYMPTOMATIC | POSITIVE | POSITIVE | NEGATIVE | NEGATIVE | NEGATIVE |
| 143A-129 | A | SYMPTOMATIC | POSITIVE | POSITIVE | PRESUMPTIVE POSITIVE | NEGATIVE | POSITIVE |
| 189A-112 | B | ASYMPTOMATIC | NEGATIVE |  | NEGATIVE | NEGATIVE | NEGATIVE |
| 189A-153 | B | ASYMPTOMATIC | NEGATIVE | NEGATIVE | NEGATIVE | INCONCLUSIVE | NEGATIVE |
| 199A-271 | A | ASYMPTOMATIC | NEGATIVE | NEGATIVE | NEGATIVE | INCONCLUSIVE | NEGATIVE |
| 199A-275 | A | SYMPTOMATIC | POSITIVE | NEGATIVE | NEGATIVE | INCONCLUSIVE | NEGATIVE |
| 202A-008 | B | ASYMPTOMATIC | NEGATIVE | NEGATIVE | NEGVATIVE | INCONCLUSIVE | NEGATIVE |

**Table S5** PPA for PIS+ subjects for different Ct thresholds

| Cohort A, All Subjects | | | | |
| --- | --- | --- | --- | --- |
|  | PIS+ All Ct | Ct<30 | Ct<33 | Ct<36 |
| IDNOW Pos | 294 | 191 | 230 | 269 |
| IDNOW Neg | 41 | 2 | 7 | 23 |
| Total | 335 | 193 | 237 | 292 |
| PPA (95%CI) | 87.8 (83.8, 91.1) | 99.0 (96.3, 99.9) | 97.0 (94.0, 98.8) | 92.1 (88.4, 94.9) |
| Cohort A, Symptomatic Subjects | | | | |
|  | PIS+ All Ct | Ct<30 | Ct<33 | Ct<36 |
| IDNOW Pos | 237 | 166 | 195 | 221 |
| IDNOW Neg | 17 | 1 | 5 | 9 |
| Total | 254 | 167 | 200 | 230 |
| PPA (95%CI) | 93.3 (89.5, 96.1) | 99.4 (96.7, 100.0) | 97.5 (94.3, 99.2) | 96.1 (92.7, 98.2) |
| Cohort A, Asymptomatic Subjects | | | | |
|  | PIS+ All Ct | Ct<30 | Ct<33 | Ct<36 |
| IDNOW Pos | 40 | 21 | 26 | 35 |
| IDNOW Neg | 18 | 1 | 2 | 10 |
| Total | 58 | 22 | 28 | 45 |
| PPA (95%CI) | 69.0 (55.5, 80.5) | 95.5 (77.2, 99.9) | 92.9 (76.5, 99.1) | 77.8 (62.9, 88.8) |
| Cohort B, Asymptomatic Subjects | | | | |
|  | PIS+ All Ct | Ct<30 | Ct<33 | Ct<36 |
| IDNOW Pos | 34 | 16 | 19 | 26 |
| IDNOW Neg | 7 | 2 | 3 | 5 |
| Total | 41 | 18 | 22 | 31 |
| PPA (95%CI) | 82.9 (67.9, 92.8) | 88.9 (65.3, 98.6) | 86.4 (65.1, 97.1) | 83.9 (66.3, 94.5) |

Cohort A: subjects with suspected COVID-19 infection (with or without symptoms); Cohort B: asymptomatic subjects with no reason to suspect COVID-19 exposure.
